# Inflammatory Markers in Severity of Intracerebral Hemorrhage II: A Follow Up Study

**DOI:** 10.7759/cureus.12605

**Published:** 2021-01-10

**Authors:** Jacob E Bernstein, Jonathan D Browne, Paras Savla, James Wiginton, Tye Patchana, Dan E Miulli, Margaret Rose Wacker, Jason Duong

**Affiliations:** 1 Neurosurgery, Riverside University Health System Medical Center, Moreno Valley, USA; 2 School of Medicine, California University of Science and Medicine, Colton, USA; 3 Neurosurgery, Arrowhead Regional Medical Center, Colton, USA

**Keywords:** intracerebral hemorrhage, c-reactive protein, tnf alpha, homocysteine, vegf angiogenesis

## Abstract

Introduction

Spontaneous intracerebral hemorrhage (ICH) results in significant morbidity and mortality. The pathogenesis of brain injury after ICH is thought to be due to mechanical damage followed by ischemic, cytotoxic, and inflammatory changes in the underlying and surrounding tissue. Various inflammatory and non-inflammatory biomarkers have been studied as predictors and potential therapeutic targets for intracerebral hemorrhage. Our prior study showed an association with low vascular endothelial growth factor (VEGF) levels and increased mortality. This current study looks to expand on our prior results and will look at the relationship between tumor necrosis factor alpha (TNFα), C-reactive protein (CRP), VEGF, Homocysteine (Hcy), and CRP to albumin ratio (CAR) in predicting outcomes and severity in spontaneous intracerebral hemorrhage.

Methods

We conducted a retrospective chart review of patients with spontaneous intracerebral hemorrhage with TNFα, CRP, VEGF, Hcy levels drawn on admission. Albumin and CRP levels on admission were used to calculate CAR. Ninety-nine patients were included in the study. Primary outcomes included death, early neurologic decline (END), and hemorrhage size. Secondary outcomes included late neurologic decline (LND), Glasgow Coma Scale (GCS) on admission, GCS on discharge, ICH score, change in hemorrhage size, need for surgical intervention, and length of ICU stay.

Results

A total of 99 patients were included in this study, with 42% requiring surgical intervention and an overall mortality of 16%. Basal ganglia hemorrhage was seen in 41% of patients. Hcy and CAR were significantly correlated with ICH size in basal ganglia patients (r-=0.36, p=0.03; r=0.43, p=0.03, respectively). CAR was significantly correlated with ICH score (r=0.33, p=0.007874). Admission VEGF levels less than 45 pg/ml had 8.4-fold increase in mortality (odds ratio [OR] 8.4545, p=0.0488). Patients with TNFα levels greater than 1.40 pg/ml had a 4.1-fold increase in mortality (OR 4.1, p=0.04)

Conclusion

Our study demonstrated that low levels (<45 pg/ml) of VEGF were associated with an 8.4-fold increase in mortality, supporting the neuroprotective effect of this protein. Elevated Hcy and CAR levels were associated with an increase in hemorrhage size in patients with basal ganglia hemorrhages. TNFα levels greater than 1.40 pg/ml were associated with a 4.1-fold increase in mortality, and this together with CAR being correlated with increased hemorrhage size and ICH score further demonstrate the inflammatory consequences after intracerebral hemorrhage. Future studies directed at lowering CRP, TNFα, and Hcy and/or increasing VEGF in intracerebral hemorrhage patients are needed and may be beneficial.

## Introduction

Spontaneous intracerebral hemorrhage (ICH) accounts for 10-20% of all strokes and has a higher morbidity and mortality than ischemic stroke, with 30-day mortality rates of 37-52% [1,2]. Medical management is the primary treatment modality and consists primarily of airway protection, oxygenation, tight blood pressure control, and correction of coagulopathy. Surgical intervention has limited efficacy compared to medical management and is often reserved for large hemorrhages causing mass effect or for intraventricular hemorrhage causing hydrocephalus. The pathogenesis of brain injury after ICH is thought to be due to mechanical damage followed by ischemic, cytotoxic, and inflammatory changes in the underlying and surrounding tissue [1,2]. There has been much interest in utilizing biomarkers in order to predict outcomes and hopefully develop new therapies for stroke prevention and management. Biomarkers such as tumor necrosis factor alpha (TNFα), C-reactive protein (CRP), homocysteine (Hcy), and vascular endothelial growth factor (VEGF) have previously been associated with differential findings in ischemic and hemorrhagic stroke [3-21]. Our prior investigation into these markers in 2018 found an association between low VEGF and increased mortality. There was also a non-significant trend in hemorrhage size and mortality with elevated levels of CRP and homocysteine. However, that study was limited secondary to its small size [21]. In addition to the previous markers, this study will also look at the association of C-reactive protein/albumin ratio (CAR) which is an inflammation-based index used in predicting outcomes in a variety of illnesses (sepsis, small cell lung cancer, pancreatic cancer, etc) and has recently been found to be an independent risk factor for mortality in patients with traumatic brain injury, ischemic stroke, and ICH [22-24]. We look to further investigate the relationship between CAR and the severity of intracerebral hemorrhage.

## Materials and methods

Study design

We conducted a retrospective review of a prospectively collected database of patients admitted to the neurosurgery service at Arrowhead Regional Medical Center from 2015 to 2020 with a diagnosis of spontaneous ICH or intraventricular hemorrhage (IVH). In addition to the standard of care, patients had the following labs drawn on admission: TNFα, CRP, Hcy, and VEGF. CAR was calculated in patients with CRP and albumin drawn on admission. Patients were included in the study if older than age 18, had a diagnosis of spontaneous intracerebral or intraventricular hemorrhage without underlying trauma, vascular abnormality, or evidence of hemorrhagic transformation of ischemic stroke, and if they had at least one of previously mentioned biomarkers drawn on admission for intracerebral hemorrhage. A total of 99 patients were included in the study. Primary outcomes included death, early neurologic decline (END), and hemorrhage size. Secondary outcomes included late neurologic decline (LND), Glasgow Coma Scale (GCS) on admission, GCS on discharge, ICH score, change in hemorrhage size, need for surgical intervention, and length of intensive care unit (ICU) stay.

All patients were admitted to ICU for a minimum of 24 hours with neurological checks every hour. Patients were intubated at the discretion of emergency department or if GSC<=8. Systolic blood pressure was strictly maintained with the goal of <140 mmHg. External ventricular drain (EVD) placement occurred if GCS <=8, extensive intraventricular involvement, symptomatic hydrocephalus, or need for use of intraventricular tissue plasminogen activator (t-PA) administration. Some patients underwent bedside intraparenchymal hematoma evacuation and drain placement or craniotomy versus craniectomy, depending on size of hemorrhage, midline shift, patient’s clinical exam, and clinician discretion. Repeat CT head was obtained six to eight hours from initial CT scan to evaluate for change in the size of hematoma. Volume of hemorrhages was calculated via ABC/2 formula [25]. A single neurosurgery resident calculated hemorrhage volume for initial and repeat CT scan. Vascular studies were obtained if underlying vascular abnormalities were suspected. TNFα, CRP, Hcy, and VEGF levels were determined on admission for hemorrhagic stroke. The labs were performed via Quest Diagnostics. Early neurologic decline was defined as a decrease in GCS of 2 or more points within 48 hours of admission. Late neurologic decline was defined as a decrease of 2 or more GCS points after 48 hours after admission.

Statistical analysis

Descriptive statistics were presented as frequencies and proportions for categorical variables. Chi-squared, odds ratio (OR), and Pearson correlation coefficient were used in analyzing the data. All statistical tests were two-sided. P-values <0.05 were considered to be statistically significant.

## Results

A total of 99 patients were included in this study, with 42% requiring surgical intervention, including EVD placement, and an overall mortality of 16%. The surgical procedures performed, and patient demographics are listed in Table 1. Early neurological decline was observed in 10 patients and late neurological decline was seen in 13 patients with both being significantly correlated with mortality (Table 2). The most common hemorrhage occurred in the basal ganglia (41%) as seen in Table 1. GCS on admission was negatively correlated with mortality, while ICH score, presence of IVH, initial ICH size, repeat ICH size, change in ICH size, END, LND, midline shift, and presence of surgical procedure were all positively correlated with mortality (Table 2). None of the four markers or the CAR was significantly correlated with mortality although TNFα and VEGF had weak non-significant correlations (Table 2). With regards to ICH size, TNFα, Hcy, and CAR were all weakly correlated with ICH size although this was not significant (r=0.12, r=0.12, r=0.20, respectively). There was a weak negative correlation between VEGF and ICH size although this was not significant (r=-0.18). TNFα and VEGF were mildly correlated with hemorrhage size in basal ganglia patients, although non-significantly (r=0.32, p=0.08; r=-0.21, p=0.26, respectively), while Hcy and CAR were significantly correlated with ICH size in basal ganglia patients (r=-0.36, p=0.03; r=0.43, p=0.03, respectively). There was no significant correlation between the markers in ICH size in lobar hemorrhage, although TNFα was mildly correlated (r=0.31, p=0.12).

**Table 1:** Patient demographics, ICH statistics, and surgical interventions GCS=Glasgow Coma Score ICH=Intracerebral Hemorrhage ICU=Intensive Care Unit

Number of Patients	99
Male/Female	54/45
Age (range)	61.8 (21-88)
GCS on admission (range)	11.9 (3-15)
ICH Score (30 day mortality): 0 (0%), 1 (13%), 2 (26%), 3 (72%), 4 (97%), 5 (100%)	0=26 (3.8% mortality) , I=39 (7.6% mortality), 2=15 (20% mortality), 3=7 (14.3% mortality), 4=11 (64% mortality), 5=1 (100% mortality)
ICH Location	Basal ganglia(41 patients), Lobar (35 patients), Thalamus (10 patients), Cerebellum (6 patients), Caudate (3 patients), Brainstem (2 patients), Intraventricular only (2 patients)
Length of ICU Stay	6.02 days
Early Neurological Decline	10/99
Late Neurological Decline	13/99
Mean Intracerebral Hemorrhage Volume (ml)	Initial-25.3 ml , Repeat-23.8 ml, Change-1.5 ml
Presence of Intraventricular Hemorrhage	37/99
Number of Procedures	42 procedures:26 external ventricular drain, 9 intraparenchymal drains, 2 craniectomies, 5 ventriculoperitoneal shunts
Midline shift (mm)	2.3
Mortality	16/99

**Table 2:** Factors with correlation to patient mortality ICH=intracerebral hemorrhage CAR=CRP:albumin ratio VEGF=vascular endothelial growth factor CRP=C-reactive protein GCS=Glasgow coma scale TNFα=tumor necrosis factor alpha

Factors and Mortality	Pearson (r)	P value (significance <0.05)
Age	0.09	0.325
GCS on admission	-0.35	0.00041
Early Neurological Decline	0.31	0.00019
Late Neurological Decline	0.56	<0.00001
ICH Score	0.47	<0.00001
Intraventricular Hemorrhage	0.23	0.023
Initial ICH Size	0.47	<0.00001
Repeat ICH Size	0.39	0.000048
Change in ICH Size	0.29	0.0029
Midline shift	0.57	<0.00001
TNFα	0.15	0.197
Homocysteine	0.11	0.379
CRP	0.022	0.835
VEGF	-0.15	0.178
CAR	0.022	0.859

**Figure 1:**
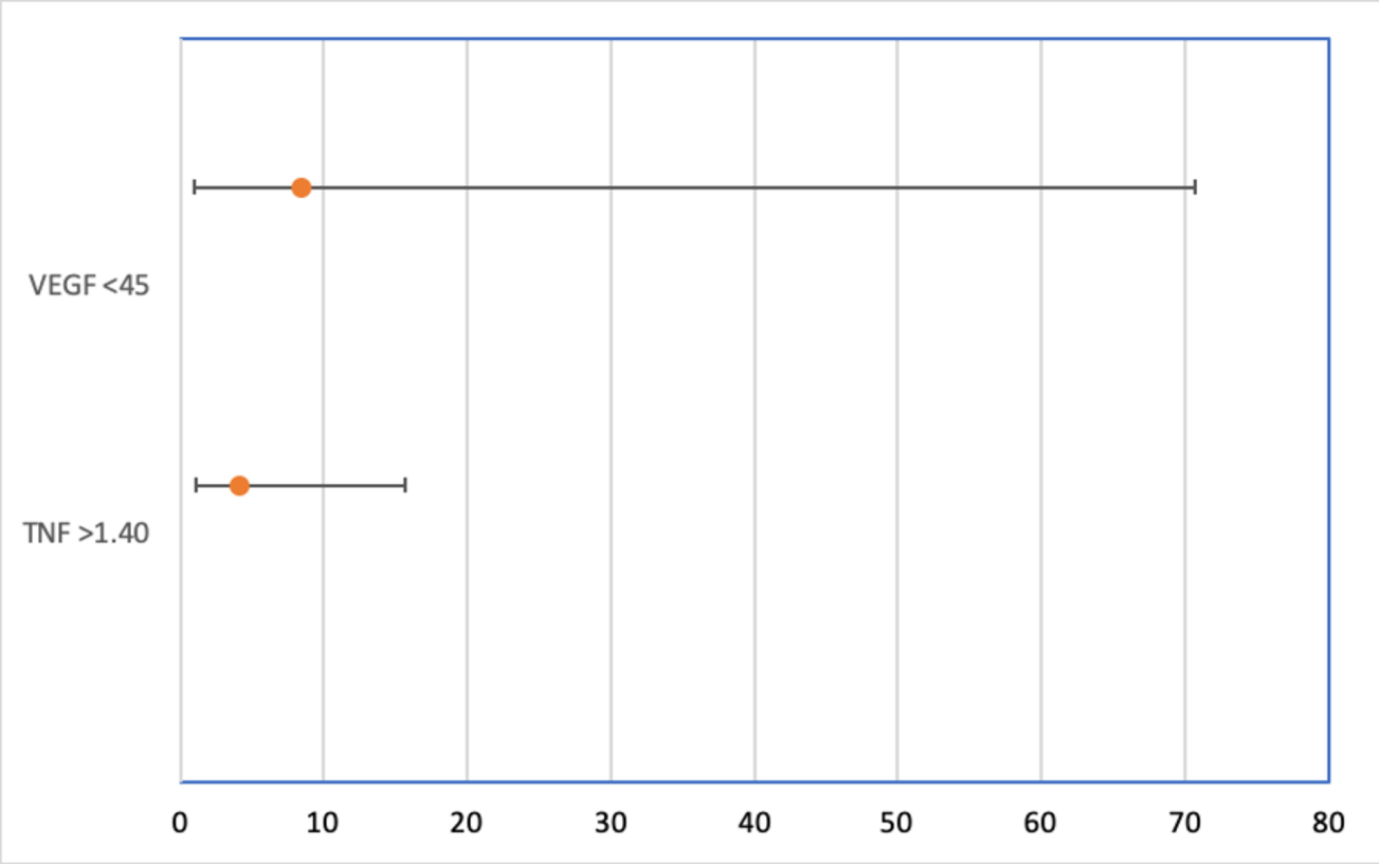
Odds ratios with 95% confidence intervals VEGF=Vascular endothelial growth factor TNF=Tumor necrosis factor

CAR was significantly correlated with ICH score (r=0.33, p=0.007874). Patients with admission VEGF levels less than 45 pg/ml had 8.4-fold increase in mortality (OR 8.4545, p=0.0488) (Figure 1). Patient with TNFα levels greater than 1.40 pg/ml had a 4.1-fold increase in mortality (OR 4.1, p=0.04) (Figure 1). There were no significant correlations between the biomarkers tested and END, LND, GCS on admission, GCS on discharge, length of ICU stay, or need for surgical procedure. Four patients were found to have a new hemorrhage during admission, with two of these as a complication of EVD placement. Both patients with post EVD placement hemorrhages died. The two remaining patients with new hemorrhages lived. One patient had a recurrent hemorrhage two months after initial presentation and died.

## Discussion

Our study demonstrated that low levels of VEGF (<45 pg/ml) on admission significantly increased mortality by 8.4-fold. This finding adds to our previous study showing that low levels were associated with mortality [21]. It is also consistent with Sobrino et al. (2009), which showed that high levels (>330 pg/ml) of VEGF at 72 hours were associated with improved outcome in patients with intracerebral hemorrhage. This indicates that VEGF is neuroprotective in patients with intracerebral hemorrhage, which is further supported by findings in prior animal studies [26]. In a mouse study, VEGF-induced stem cell administration has been shown to increase angiogenesis and result in improved functional outcome [26]. This increase in angiogenesis likely reduces cerebral ischemia in intracerebral hemorrhage as it has been shown to in ischemic stroke rat studies [27]. In one rat study, intracerebroventricular injection of VEGF was shown to increase angiogenesis and decrease cerebral ischemia while intravenous injection of VEGF increased cerebral infarct volume [27]. Currently, there are no published studies on administration of VEGF or induction of VEGF during intracerebral hemorrhage in humans. This is a promising potential therapeutic modality that could be applied to intracerebral hemorrhage and warrants further research.

TNFα is a pro-inflammatory cytokine release by neuronal macrophages, astrocytes, and microglial cells during intracerebral hemorrhage. This leads to increased release of oxidase and proteases that result in secondary brain injury [3,28]. Prior studies demonstrate that TNFα is associated with worse outcome and mortality in intracerebral hemorrhage [2,6,20]. Our study found TNFα levels to be mildly correlated with ICH size, but this was not significant. However, patients with a TNFα level on admission greater than 1.40 pg/ml were found to have a 4.1-fold increase in mortality. Potential treatments for intracerebral hemorrhage aimed at reducing TNFα levels include anti-TNFα monoclonal antibodies and atorvastatin [26]. Blocking endogenous TNFα via monoclonal anti-TNFα antibodies in rats has been shown to decrease cerebral ischemia [29]. Ewen et al. (2013) demonstrated that atorvastatin induced a dose-dependent reduction of TNFα in plasma and brain tissue in rats with intracerebral hemorrhage and an increase in interleukin 10 (IL-10) which has anti-inflammatory functions. This resulted in a decrease in hemispheric water content, decrease infiltration of leukocytes and microglia, and improvement in neurological deficits among rats treated with atorvastatin [28]. Statin in therapy in ischemic stroke prevention and treatment is well documented in the literature while the benefits of continuation or initiation of statins after ICH is still being investigated [30]. Our study supports the initiation and continuation of statin therapy in hemorrhagic stroke. Once statin therapy is initiated, longer-term follow up could study the effects on functional outcome, recurrence of hemorrhagic stroke, and rates of development of any kind of stroke in the future.

Hcy is not released due to intracerebral hemorrhage but as a result of genetic conditions, vitamin B deficiencies, smoking, high methionine diet, and sedentary lifestyles [11]. Elevated levels have been shown to increase risk of hemorrhage stroke [12]. Our prior study showed a trend toward elevated homocysteine and increase hemorrhage size while our current study confirmed that elevated levels of homocysteine are associated with larger basal ganglia hemorrhages. This was also demonstrated in other prior studies [13,21]. Lowering homocysteine should be targeted at preventing hemorrhagic strokes and should be checked routinely, especially in individuals with significant risk factors for stroke. Dietary supplementation with folic acid, vitamin B6, and vitamin B12 and lifestyle modifications such as compliance with blood pressure medications, maintaining a regular exercise schedule, smoking cessation, and a low methionine diet have been shown to decrease stroke risk [11-13].

CRP is increased in response to acute inflammation, leads to cell apoptosis, and has been shown in previous studies to be a predictor of mortality and associated with worse outcomes after intracerebral hemorrhage [9,10,22]. In our study, CRP was the most commonly elevated of the markers (50% of patients tested) but only showed a mild non-significant correlation with ICH score. However, our study failed to show any relationship between elevated CRP and our primary or secondary outcomes. In order to look further at the relationship of CRP with intracerebral hemorrhage, we evaluated the CAR, which has been used in predicting outcomes in a variety of illnesses (sepsis, small cell lung cancer, pancreatic cancer, etc) and has recently been found to be an independent risk factor of mortality in patients with traumatic brain injury and ischemic stroke [22,23]. This ratio reflects the combined effects of nutrition and inflammation with evelated CAR representing increased inflammation with low nutrition status [22]. Our study demonstrated that CAR was moderately correlated with ICH score and hemorrhage size in patients with basal ganglia hemorrhages but not with mortality. However, both ICH score and hemorrhage size were independently correlated with mortality. Only one other study has evaluated CAR in intracerebral hemorrhage. Bender et al. (2020) reviewed 379 patients with intracerebral hemorrhage and found that CAR levels greater than 1.22 were significantly associated with mortality [24]. Optimizing nutritional status in patients with ICH in addition to CRP lowering therapy (vitamin C, angiotensin-converting enzyme inhibitors, cyclooxygenase inhibitors, etc) is a potential way to mitigate these effects or poses a potential therapeutic avenue to be studied [21]. Further studies into modulating CAR after the onset of hemorrhagic stroke can be studied to evaluate the utility of modifying CRP and albumin in an inpatient setting, rather than only as a preventative measure.

Limitations

One of the biggest limitations of our study is that patients presenting to our institution with intracerebral hemorrhages deemed “catastrophic” or where family elect to pursue “comfort” measures in the emergency department are not admitted to the neurosurgery service. In these patients, the above biomarkers were not drawn by the emergency department or admitting medical service. Thus, our study missed the opportunity to evaluate these biomarkers in some patients with the most severe intracerebral hemorrhages. Another limitation is that not all of the biomarkers and the CAR were available for all of the 99 patients as TNFα was available in 71 patients, Hcy in 72 patients, CRP in 85 patients, VEGF in 74 patients, and CAR in 63 patients. As many of these laboratory markers are send-out studies, it is difficult to ensure that admission blood samples are both sent and tested appropriately; samples could be lost in transit, deemed insufficient, or have other issues resulting in lack of results. This could have limited the effectiveness of our study to obtain associations with these markers. Lastly, there was no long-term follow-up after patients were discharged from the hospital. Developing a follow-up system in conjunction with our internal and family medicine colleagues could mitigate this issue, along with providing better overall medical care for patients.

## Conclusions

Our study demonstrated that low levels (<45 pg/ml) of VEGF were associated with an 8.4-fold increase in mortality, supporting the neuroprotective effect of this protein. Elevated Hcy and CAR levels were associated with an increase in hemorrhage size in patients with basal ganglia hemorrhages. TNFα levels greater than 1.40 pg/ml were associated with a 4.1-fold increase in mortality, and this together with CAR being correlated with increased hemorrhage size and ICH score further demonstrate the inflammatory consequences after intracerebral hemorrhage. Future studies directed at lowering CRP, TNFα, and Hcy and/or increasing VEGF in intracerebral hemorrhage patients are needed and may be beneficial. Furthermore, studies aimed at lowering these values in high-risk patients prior to intracerebral hemorrhage should also be evaluated as a potential preventative measure.
